# Role of Chimeric Antigen Receptor T-Cells in the Evolving Therapeutic Landscape of Multiple Myeloma: A Literature Review

**DOI:** 10.7759/cureus.80068

**Published:** 2025-03-05

**Authors:** Marya Radhi, Dalal A Yusuf, Ghufran M AlSaffar, Zainab A Toorani

**Affiliations:** 1 General Medicine, Royal College of Surgeons in Ireland (RCSI) - Medical University of Bahrain, Busaiteen, BHR; 2 General Surgery, Salmaniya Medical Complex, Manama, BHR; 3 Medicine, Royal College of Surgeons in Ireland (RCSI) - Medical University of Bahrain, Busaiteen, BHR; 4 Pathology, Salmaniya Medical Complex, Manama, BHR

**Keywords:** car-t, car-t therapy, chimeric antigen receptor t cell, chimeric antigen receptor t cell therapy, multiple myeloma

## Abstract

Multiple myeloma (MM) is a quotidian malignant disorder of plasma cells. It is hallmarked by the uncontrolled growth of bone marrow plasma cells, leading to multiorgan dysfunction. Despite ongoing advancements in remedies, including chemotherapy, radiotherapy, immunomodulatory drugs, stem cell transplantations, and countless other lines of treatment, the management of MM remains a challenge.

Recent studies indicate promising outcomes with chimeric antigen receptor T-cell (CAR-T) therapy, especially in patients who have previously undergone other treatments. This literature review aims to explore various facets of MM and the progress made with CAR-T therapy, emphasizing the mechanism, effectiveness, and safety profile.

## Introduction and background

Overview of multiple myeloma (MM)

MM is the most encountered hematological malignancy with low overall survival rates [1]. It arises in the plasma cells, causing the accumulation of monoclonal paraproteins, resulting in bone destruction and leading to end-organ damage [2]. Classical manifestations of MM include hypercalcemia, anemia, renal failure, recurrent bacterial infections, lytic bone lesions, and extramedullary soft tissue plasmacytomas [3]. While the incidence of the disease is increasing, it is still considered to be a hard-to-cure disease. Despite available treatment options like immunomodulatory drugs (IMiDs), proteasome inhibitors (PIs), and other monoclonal antibodies, the disease tends to recur and relapse eventually, which further reduces the prognosis [4]. MM causes substantial morbidity and mortality rates, which necessitates further research to find a solution for this disease [1,3].

Recently, chimeric antigen receptor T-cell (CAR-T) therapy has shown its efficacy by improving patient outcomes and increasing their overall survival rates [5]. It is also worth noting that the most frequent countries that used CAR-T in the studies we’ve referred to are China and the USA.

Overview of CAR-T therapy

Chimeric antigen receptors (CARs) are engineered immune cells designed with proteins that have been studied for their role in treating various cancers [5]. The structure of CARs comprises four main components: an extracellular target-binding region, a hinge region, a transmembrane domain, and one or more intracellular signaling domains. CAR-T cells are made from the patient's own body by collecting blood, and then, through a process known as apheresis, the T cells are extracted and undergo genetic engineering to express CAR on the surface of the cell; this will help to recognize the target antigen. While CAR-T therapy shows promise for MM, overcoming obstacles such as antigen escape, CAR-T cell trafficking, tumor infiltration, and potential toxicities should be considered [5,6].

The function of CARs mainly depends on their structural composition. Moreover, the location of their domain is what indicates its functionality, whether located on the extracellular, intracellular, or transmembrane regions of the dependent cell. Any mutation to their formulation may result in a different function, such as changes to their co-stimulatory domains and checkpoints when certain additives are introduced into the formation of such receptors [7].

The Food and Drug Administration (FDA) has approved CAR-T using B cell maturation antigen (BCMA) as a target in treating refractory/relapsed MM, primarily as a neoadjuvant or bridging treatment for patients preparing for hematopoietic stem cell transplantation (HSCT). The two currently available CAR-T therapies are ciltacabtagene autoleucel, or cilta-cel (Carvykti) and idecabtagene autoleucel (Abcema). Cilta-cel is approved for use after four or more prior lines of therapy, including a PI, an IMiD, and an anti-CD38 monoclonal antibody [5,8]. Most studies focused on BCMA as the principal antigen in relapsed/refractory MM [9]. Recent trials have investigated the use of CD-19 along with anti-BCMA for MM treatment and have achieved promising outcomes, which will be discussed further in the upcoming paragraphs. It is important to note that CD-19 is approved for use in certain types of lymphoma and leukemia and is still under investigation for MM [10-12].

## Review

Methodology

Several databases were used to conduct our literature review; this was done to ensure a high-quality review. The selected articles were initially searched in the following databases: PubMed, Web of Science, ScienceDirect, Scopus, and ClinicalTrials.gov, using the terms: "multiple myeloma," "multiple myelomas," "chimeric antigen receptor T-cell (CAR-T)," and "CAR-T therapy."

Inclusion criteria were that all articles be in the English language; clinical trials, case-control studies, retrospective studies, and prospective studies were included. Articles in languages other than English were excluded, as well as editorials and reviews.

Aim of the study

Through this article, we aim to provide healthcare professionals with a clear idea of how CAR-T therapy is an effective treatment for MM, as well as compare it to existing treatment methodologies and also discuss its limitations and adverse effects. Knowing the recent advancements in MM treatment will improve patient outcomes and enhance current treatment protocols.

Multiple myeloma (MM)

MM is recognized as the predominant primary bone malignancy, particularly prevalent in the African American population in the United States, where the incidence is twice that of Caucasians. The median diagnosis age ranges from 40 to 70 years, with a slight male predominance, having a ratio of 1.4:1 [1,2].

While the incidence of MM is rising, the disease remains incurable; however, CAR-T therapy has demonstrated encouraging results, which provides hope to the MM patients who have undergone multiple treatment lines before opting for CAR-T [1,13]. The incidences of MM in the Gulf countries have been illustrated in Table 1 [14].

**Table 1:** Number of cases and deaths of multiple myeloma (MM) within the Gulf Cooperation Council (GCC) in 2022 Table credit: [14]

Country	Bahrain	Kuwait	KSA	UAE	Oman
Number of cases	15	65	295	69	61
Number of deaths	12	44	229	37	47

Pathophysiology of MM

The development of MM begins with monoclonal gammopathy of undetermined significance (MGUS), an asymptomatic condition that can evolve into MM or related disorders [14]. Studies hypothesize that MGUS is present in all patients with MM many years prior to its development. The specific reasons and pathway by which MGUS develops into MM are still unknown, but there might be aspects that contribute to its development, such as genetic changes that cause a higher expression of promoter genes, leading to increased plasma cell proliferation [2].

Etiology of MM

The etiology of MM is not fully known; however, it can be multifactorial, including genetics, radiation exposure, chronic inflammation, and infections. Regarding genetics, alterations in genes, especially on chromosome 14, are associated with a risk of developing MM. In addition, myeloma cells have an abnormal expression of antigens, including CD-56, CD-38, CD-138c, and CD-19 [2]. There are some oncogenes (KRAS, NRAS, BRAF) that may contribute to plasma cell proliferation in the disease. Moreover, the presence of certain oncogenes and high-risk chromosomal abnormalities can increase the likelihood of developing MM. For example, p53 mutations, del(17p), t(14;16), t(14;20), t(4;14), or gain of 1q. The presence of these mutations is presumed to be an elevated risk. The presence of any two high-risk factors is classified as double-hit myeloma; three or more are thought to be triple-hit myeloma [13].

Morphology of MM

There are various morphologies of plasma cells in MM, such as mature large cells (two to three times larger than a lymphocyte cell), eccentric nuclei, basophilic cytoplasm, a perinuclear halo due to the Golgi apparatus, and Mott cells that appear as clustered cytoplasmic droplets. However, plasma cells in MM can sometimes have immature features, such as a high nuclear-to-cytoplasmic ratio, larger cell size, and loose chromatin. These features are particularly seen in relapsed forms of MM. Other types of unusual morphologies include multinucleated cells or fiery red cytoplasm, known as flame cells, which are observed in recurrent MM. 

Some of the other morphological features of a myeloma cell that can be seen are Russel bodies, giant cells, signet cells, storage cells, Dutcher bodies, Pseudo-Auer rods, and Snapper-Schneid granules.

Research shows that some of the morphological features of nuclei contribute to the poor prognosis in the setting of MM. These features include the size and shape of the nuclei, as well as the number of nucleoli [2,15].

Clinical Variants of MM

There have been no studies conducted so far on the effect of treating smoldering or non-secretory MM patients with CAR-T therapy. However, there was only one study that showed the response of just two patients who developed primary plasma cell leukemia (PCL). One of those patients achieved a full response, while the second patient had a good partial response [16-19]. The clinical variants of MM are shown in Table 2.

**Table 2:** Various clinical variants of multiple myeloma

Variants	Description	References
Smoldering multiple myeloma (asymptomatic)	M-protein >2 g/dL, clonal bone marrow plasma cells (PCs) >20%, and serum immunoglobulin free light chains (FLC) ratio >20; Progression to multiple myeloma (MM) is 23-82%, depending on the severity; Management is by observation until progression to MM	[17]
Non-secretory multiple myeloma	Clonal bone marrow plasma cells ≥10% or biopsy-proven plasmacytoma, evidence of end-organ damage; Absence of serum and urinary monoclonal protein on electrophoresis and immunofixation	[18]
Plasma cell leukemia (PCL)	>2 x 10^9/L peripheral plasma blood cells, or >20% of white cell counts with differentials rationalizing plasmacytosis; Primary PCL is malignant plasma cells seen in the leukemic phase, whereas Secondary PCL is the leukemic transformation of MM. Overall, it has a poor prognosis of only 7 months	[19]

Symptoms and Signs of MM

MM can be asymptomatic or symptomatic. In asymptomatic patients, it can be incidentally found through routine blood tests showing proteinuria, anemia, or hypercalcemia [20]. However, in symptomatic patients, it usually presents with localized bone pain, mainly in the spine, ribs, or as a pathological fracture. In addition, some patients may have non-specific symptoms, including but not limited to nausea, vomiting, malaise, weight loss, and recurrent infections [2].

Hypercalcemia can cause polydipsia, polyuria, abdominal pain, in addition to bone pain, and altered mental status. Renal failure leads to edema, acidosis, and electrolyte disturbances. Anemia leads to fatigue, pallor, and palpitations. Bone disease can cause fractures, reducing height due to spinal cord compression, radicular pain, and kyphosis [2].

Diagnosis and Evaluation of MM

Routine laboratory tests: The diagnosis and evaluation of MM, as per the International Myeloma Foundation, are shown in Table 3 [21].

**Table 3:** Routine laboratory tests for diagnosing multiple myeloma Table credit: [21]

Role	Tests
Assessing blood cells	Complete blood count (CBC), peripheral blood smear
Assessing kidney functions	24-hour urine collection for proteinuria, urinalysis, creatinine clearance, estimated glomerular filtration rate, and serum creatinine
Assessing proteins and other substances	Total protein test, lactate dehydrogenase, C-reactive protein, serum beta-2 microglobulin, blood glucose levels, and calcium levels
Assessing monoclonal proteins	Serum protein electrophoresis, immunofixation electrophoresis of blood or urine, serum free light chain assay, serum heavy/light chain assay, and quantitative immunoglobulins testing
Assessing bone marrow	Aspirate and trephine biopsy with testing for cytogenetics, fluorescent in situ hybridization, and immunophenotyping

Bone testing: Skeletal survey (spine, pelvis, skull, humeri, and femurs) and a low-dose whole-body CT or MRI, if there is evidence of one or more sites of osteolytic destruction (>5 mm) on CT or PET scan, confirm criteria for bone disease in MM and meet the CRAB criteria for MM.

Previously, a diagnosis of MM was made if the clonal bone marrow plasma cells were ≥10% on bone marrow biopsy (or if a biopsy-proven plasmacytoma was present), in addition to at least one of the following CRAB criteria [2]: C: Serum calcium level >0.25 mmol/L (>1 mg/dL) higher than the upper limit of normal or >2.75 mmol/L (>11 mg/dL); R: Renal insufficiency (creatinine >2 mg/dL (>177 micromole/L) or creatinine clearance <40 mL per minute); A: Anemia (hemoglobin <10 g/dL or hemoglobin >2 g/dL below the lower limit of normal); B: One or more osteolytic bone lesions on skeletal radiography, CT, or PET-CT, often described as punched-out, round, radiolucent lesions. In 2014, the International Myeloma Working Group (IMWG) added more variables that are associated with an 80% risk of developing myeloma-related organ damage [2].

Bone marrow plasma cells >60%, which can be calculated by doing a peripheral blood smear, involved/uninvolved serum free light chain ratio ≥100, and an abnormal MRI with more than one focal lesion, each >5 mm. With the updated factors, the currently used criteria are known as the SLimCRAB, where both the original CRAB and the recent IMWG criteria are added [2].

The listed elements were added to the CRAB criteria as diagnostic alternates to myeloma-defining events: bone marrow plasma cells equal to 60%, involved/uninvolved serum free light chain ratio greater than or equal to 100, and an abnormal MRI with more than one focal lesion, each greater than 5 mm, that were often missed on previous skeletal surveys. Currently, the presence of any CRAB criteria or any of the criteria mentioned above justifies diagnosis and therapy [2].

Staging of MM

Stage 1: B2M <3.5 mg/L, albumin ≥3.5 g/dL, normal lactate dehydrogenase (LDH) levels, and standard-risk cytogenetics; Stage 2: neither Stage 1 nor Stage 3; Stage 3: B2M >5.5 mg/L, high-risk cytogenetics (del(17p), and/or t(4;14), and/or t(14;16)), or elevated LDH levels [2].

Treatments of MM

There have been several improvements in the treatment of MM over the years. These include chemotherapy, as well as autologous hematopoietic stem cell transplantation (auto-HSCT), PIs, monoclonal antibodies, and IMiDs [22]. For newly diagnosed MM, the standard treatment involves induction therapy with lenalidomide (an IMiD), bortezomib (a PI), and dexamethasone (a steroid). This regimen is commonly referred to as VRd, followed by high-dose melphalan, a chemotherapy drug, in case the patient will undergo a stem cell transplant. Following the autologous stem cell transplant, maintenance therapy is initiated [23].

Monoclonal antibodies, such as daratumumab, which is a CD-38 antibody, have been approved to treat MM in combination with the aforementioned regimens. In patients not eligible for stem cell transplant, it can be used with lenalidomide and dexamethasone or with bortezomib, melphalan, and prednisone. If the patient is eligible for stem cell transplant, daratumumab is used along with bortezomib, thalidomide, and dexamethasone [24].

In the CREST trial carried out in 2008, relapsed patients or those refractory to frontline therapy were randomized to receive two different doses of bortezomib, a PI. In patients with a suboptimal response to bortezomib, dexamethasone was added. The overall survival for the low-dose group (1.0 mg/m^2^) was 26.8 months, while the higher-dose group (1.3 mg/m^2^) had 60 months. However, this result was not statistically significant (p = 0.16), as the different groups had different patient characteristics that affected the overall survival rates, such as patients' age, gender, and the number of treatments they had undergone previously. It was also noted that the addition of dexamethasone resulted in a longer overall survival period [25].

Determination of whether a patient is eligible for an autologous stem cell transplant depends on multiple factors, such as the staging of the disease, response to previous treatments, the patient's overall condition (including comorbidities), and organ function. The process of autologous stem cell transplant is similar to CAR-T therapy manufacturing and infusion. Firstly, the patient should undergo induction therapy, for which there are multiple options; however, the most effective one based on clinical trials is the four-drug regimen: daratumumab, bortezomib, lenalidomide, and dexamethasone (DVRd). After the induction process, apheresis is done to collect the stem cells from the patient's blood. Then, a high-dose therapy (HDT) is given, which involves melphalan, to reduce the tumor burden by killing the myeloma cells in the body. Two to three days after the HDT, the harvested stem cells are reinfused into the patient's blood to aid the engraftment process, where the stem cells reenter the bone marrow to produce new blood cells. Subcutaneous injections containing growth factors are also given [21].

Chimeric antigen receptor T-cell (CAR-T) therapy 

CAR-T therapy represents a novel immunotherapy strategy where the proteins that make up certain receptors are targeted against cancers, one of which is MM. They mainly target antigens such as BCMA, CS1 (SLAMF7), CD-19, GPRC5D, CD-38, NKG2DL, Kappa light chains, etc. [5]. The main antigen that has been studied extensively so far is BCMA, as it has been shown to give optimal results. Next in line is CD-19, with lesser priority given to CD-38 [5,6]. The reasoning behind BCMA having the best response is that its expression occurs in mature B cells, malignant or normal plasma cells, and plasmacytoid dendritic cells. Naive B cells, memory cells, normal hematopoietic stem cells, and other non-hematopoietic cells do not express BCMA [6]. Moreover, these cells come under the umbrella of the tumor necrosis subdivision, yet their expression is lessened in normal cells, with no expression seen in CD-34+ hematopoietic cells [26].

Other Uses of CAR-T

Using CAR-T to treat MM is not the only advantage of this revolutionary therapy. It has been proven to manage other hematological malignancies, such as relapsed/refractory acute lymphoblastic leukemia (ALL) [9]. Phase 1 human trials (NCT04196413) have been conducted on pediatric patients suffering from diffuse intrinsic pontine glioma (DIPG) and, specifically, diffuse midline gliomas (DMGs) that contained H3K27M mutations, showing promising results for its use [27].

Classification of CAR-T

CAR-T therapies are categorized into different generations according to their structures, features, limitations, etc. So far, we have come across four different components.

For first-generation cells, the single-chain fragment variable (scFV) domain is combined with both intracellular and extracellular domains, such as CD-3ζ and CD-4. Second-generation cells have co-stimulatory domains on the intracellular domains, for example, CD-28 or 4‐1BB.

**Figure 1:**
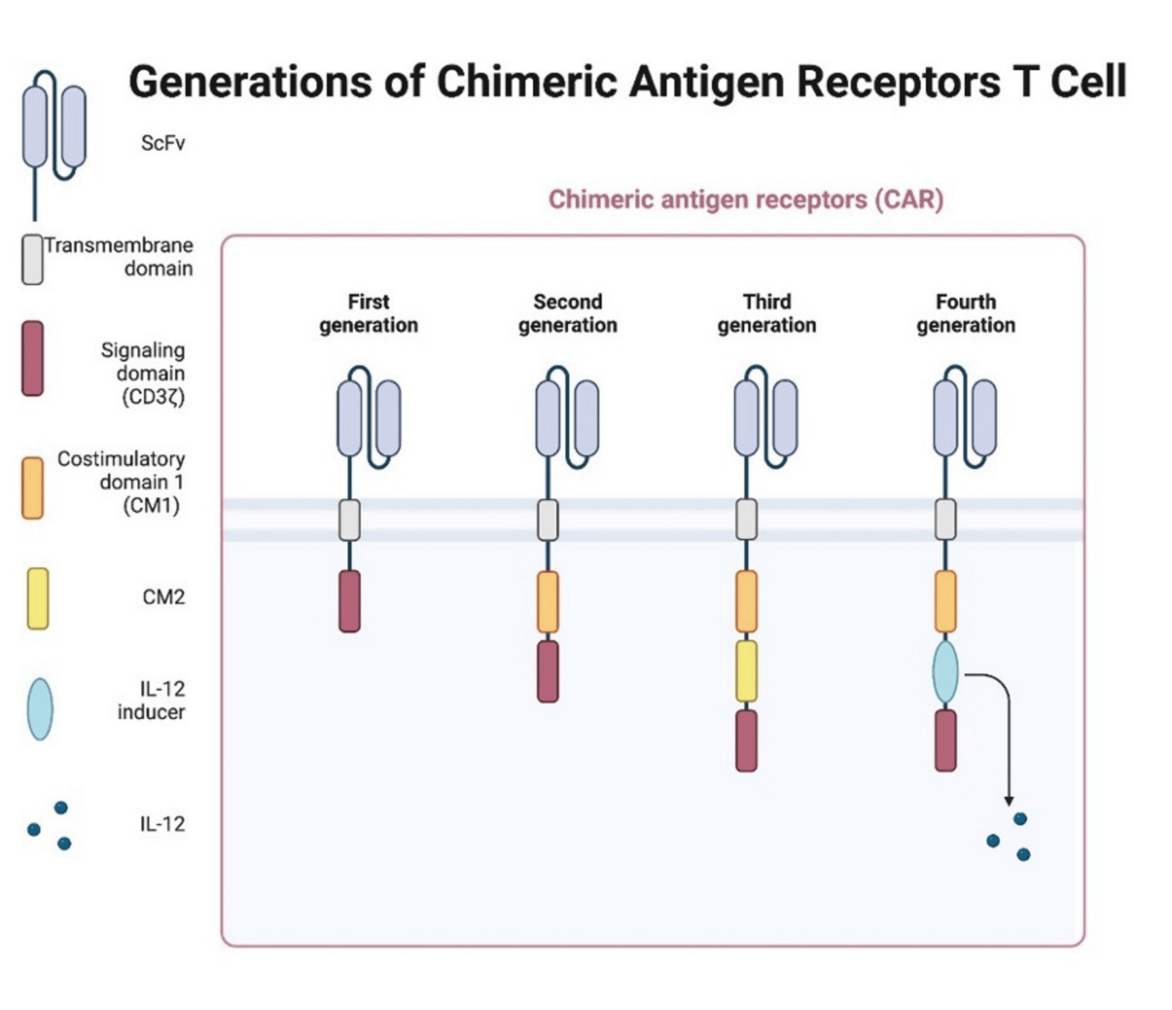
Generations of CAR-T Image credit: Created with BioRender.com CAR-T, Chimeric antigen receptor T-cell

Third- and fourth-generation CAR-T cells have a different set of costimulatory domains and present with various characteristics [28]. The first clinical trial that used fourth-generation CAR-T therapy to treat MM showed better safety precautions, as the risk of side effects was lower when compared to second-generation therapy. Yet, not many patients were tested, therefore showing certain limitations in the study itself [10,29,30]. A summary of the four generations is illustrated in Table 4 and an explanation diagram in Figure 1.

**Table 4:** Summarizes different types of CAR-T generations CAR-T, Chimeric antigen receptor T-cell

Generation	Example	Description	References
First generation	MFEζ	Poor clinical efficacy and stability	[27,28]
Second generation	BCMA-hBBz	Improve the in vivo amplification and durability of efficacy	[28,29]
Third generation	WZTL-002	Incorporate a second intracellular costimulatory domain	[10,30]
Fourth generation	BCMA-7 × 19	Co-express cytokines, such as interleukins and chemokines, or suicide genes that can substantially improve the efficacy and safety of CAR-T therapy	[29]

Indications of CAR-T

A study showed that eligible patients were chosen based on their diagnosis confirming MM, having a life expectancy of >12 weeks without signs or symptoms of active infection or other illnesses affecting the liver, heart, or other vital organs. Furthermore, they should satisfy the IMWG diagnostic criteria for relapsed/refractory MM, whilst meeting the Karnofsky performance score of ≥50 points [31]. Moreover, the patients must undergo a minimum of three major classes of MM treatment lines, including an immunomodulating agent, a PI, and an anti-CD38 monoclonal antibody, or be classified as double refractory to a PI and an IMiD. This indicates that the disease has evolved during or following treatment with the two aforementioned therapies [31-33]. Philippe Moreau has also used the same inclusion criteria for their patients to receive CAR-T therapy and has defined the endpoint as the overall response, partial or stringent complete response, similar to the IMWG [34].

Contraindications of CAR-T

Active infections and immunocompromised patients with diseases like human immunodeficiency virus (HIV) and other chronic infections like hepatitis are absolute contraindications for CAR-T therapy. Moreover, most clinical trials are still examining the safety profile of CAR-T, excluding patients with renal impairment; therefore, no precise guidelines are available yet for these patients [10,35].

Manufacturing CAR-T

A crucial part of having CAR-T as a therapy is understanding how it is engineered, as its quality and structure could affect patient care and increase the risk of side effects or response. Before patients receive CAR-T, they must undergo a few steps that take an average of two to three months for optimal results. Firstly, the patient's blood is collected and added to a centrifuge machine to separate the blood into its individual components. The white blood cells are collected, as they contain the T-cells required to generate CAR-T, while the remaining blood components are reinfused back into the patient. This process is known as leukapheresis. The T-cells collected from leukapheresis will be genetically engineered by adding a gene to the T-cells, making them express allogeneic CAR on their cell surface; they will be multiplied further to generate millions of CAR-T cells [36].

It consists of a five-step process as follows: peripheral blood mononuclear cells (PBMCs) separation and removal of red blood cells, then T-cell enrichment using paramagnetic beads (positive/negative selection), followed by transduction of T-cells with CAR genes targeting antigens, then proliferation and expansion of T-cells with recombinant interleukin 2 (IL-2) and other cytokines, and finally cryopreservation of CAR-T cells in liquid nitrogen before infusion.

**Figure 2:**
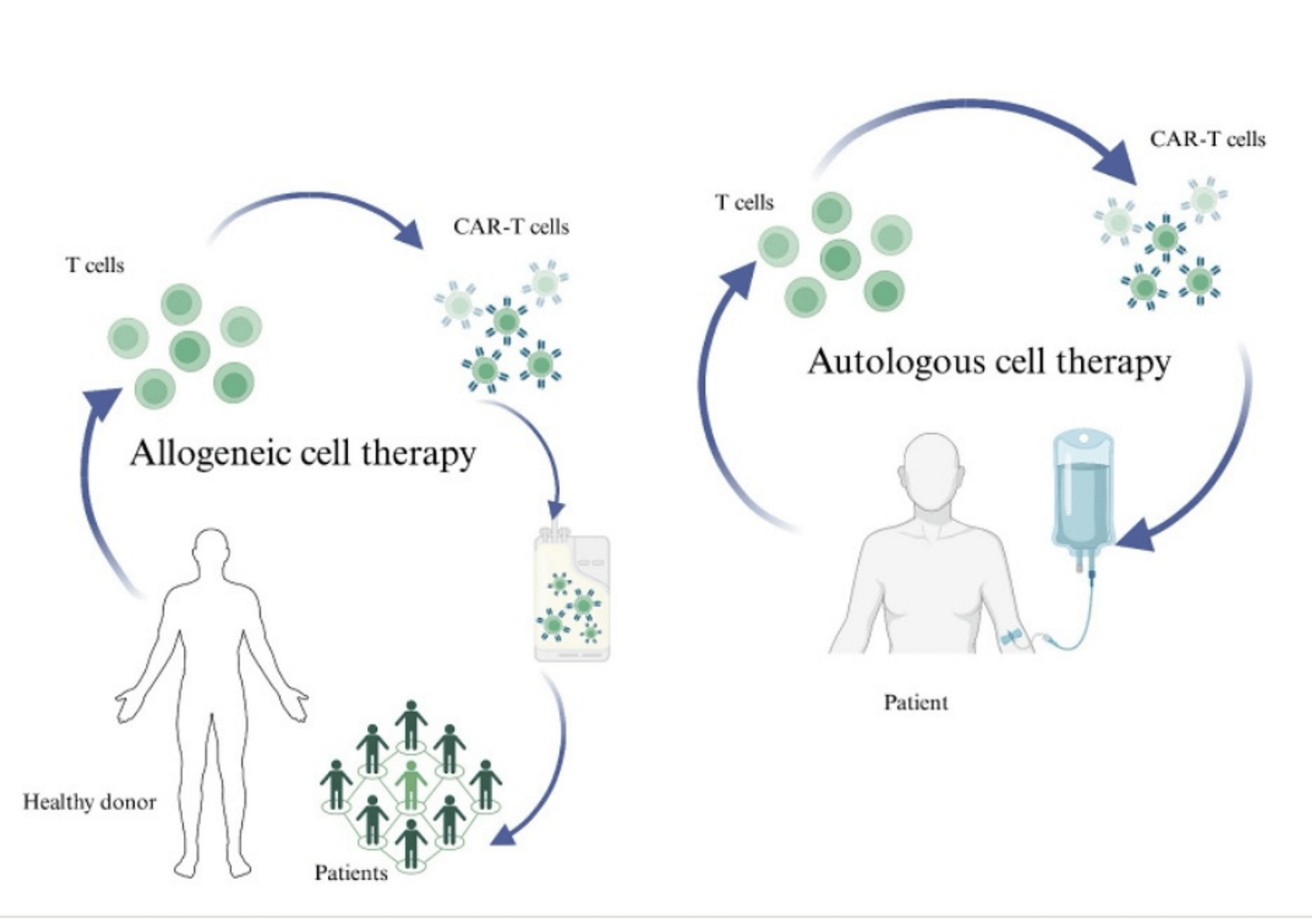
Difference between allogeneic and autologous means of cell therapy Image credit: Created with BioRender.com CAR-T, Chimeric antigen receptor T-cell

However, autologous CAR-T therapy could also be formed from one's own immune system [37]. Preclinical trials are initiated to discover the comparisons between allogeneic and autologous variants. So far, research has shown that allogeneic CAR-T therapy is favored when it comes to treatment, as autologous CAR-T therapies have long vein-to-vein times to transfuse treatment and manufacturing difficulties. Allogeneic therapies may subdue these limitations, as they make use of healthy donor cells and are engineered to restrict reactions, such as T-cell receptor (TCR)-mediated immune responses [38]. A simple description can be seen in Figure 2.

Infusion of CAR-T to the Body

Prior to infusing the CAR-T cells, the patient must undergo pre-infusion chemotherapy, usually consisting of cyclophosphamide or radiotherapy. This is to inhibit tumor growth during the period of CAR-T preparation and acts as a bridging therapy before administering CAR-T. This process is known as lymphodepletion [9,26].

**Figure 3:**
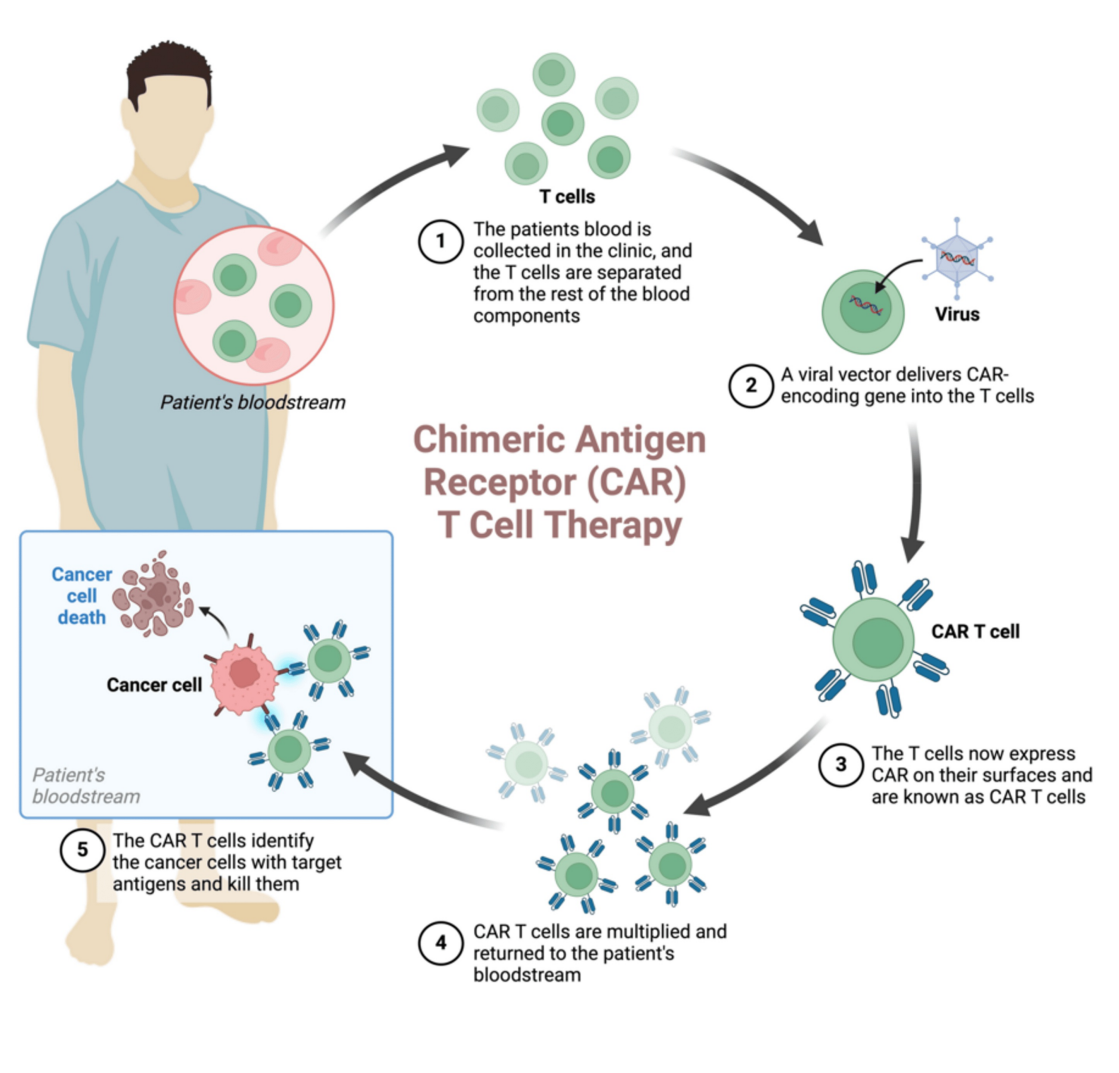
Brief explanation of how the therapy is used to treat patients Image credit: Created with BioRender.com

After these steps, the CAR-T cells have reached the stage to be administered to the patient's body. The patients may receive steroids and antipyretics to reduce the chances of having an allergic reaction and fever, respectively. It is advised that the patients remain in the hospital post-treatment for approximately 10 days to monitor the treatment outcomes and adequately manage any adverse events [28,36]. The cycle of treatment is explained using Figure 3.

If these steps are not achieved efficiently, it might affect patients' ability to receive the immunotherapy. Once the manufacturing is completed and not enough CAR-T cells are effective, patients will not be eligible for treatment [36]. Other means of increasing the response rate of CAR-T therapy would be to increase the number of targets available, while removing less differentiated cells [6].

Other factors that may cause a negative response to CAR-T therapy include antigen escape, tumor-mediated suppression, host immunity, intrinsic T-cell mechanisms, and other unspecified mechanisms, especially if the cells generated were to survive in vivo [9].

Efficacy of CAR-T

Patients undergo several treatment lines, including chemotherapy, radiation therapy, IMiDs, and even stem cell transplants, prior to opting for CAR-T. These act as a bridging therapy to prepare the body to receive CAR-T, since it is associated with several side effects, which will be addressed in the following sections [39-41]. Positive responses were associated with using CAR-T, where patients generally achieve stringent complete responses, defined as no detectable malignant plasma cells in the bone marrow, as per the MM Research Foundation, IMWG [21].

The average time to response after administering CAR-T is nearly one month; in addition, there have been positive responses in all patients, regardless of previous treatment lines. In a study by Zhao et al., involving 57 patients who were treated with LCAR-B38M CAR-T cell therapy based on body weight (0.5 × 10^6^ cells/kg), the overall response rate (ORR, defined as the percentage of patients who were able to achieve a complete response, very good partial response, or partial response after treatment) was 88%, with most of the patients (68%) achieving a complete response. Slight differences were noticed in terms of the BCMA expression. In patients with >40% BCMA expression, the ORR was 82%, while patients with <40% BCMA expression had 92%. This suggests that the lesser the expression of BCMA on the myeloma cell surface, the easier it is for the CAR-T therapy to limit the progression of the disease [26].

Another study by Shi et al. showed that after using CAR-T cell therapy along with anti-CD-19, followed by a maintenance dose of lenalidomide after autologous stem cell transplant for high-risk newly diagnosed MM patients, promising results as well as efficacy were observed. The patients were defined as high risk due to having extramedullary disease or chromosomal abnormalities. Although the number of patients was only 10, since it is a single-center study, the ORR was 100%; nine patients (90%) had a stringent complete response, and one (10%) had a complete response. During follow-up, 70% of the patients had no progression or relapse of the disease [10].

CAR-T Therapy Compared to Standard Treatment

Very few studies compared the use of CAR-T to standard care, one by San-Miguel et al., is a phase 3 trial that investigated progression-free survival by randomly assigning 419 patients to receive either cilta-cel (208 patients) or standard care (211 patients). The standard care was the physician’s choice of pomalidomide, bortezomib, and dexamethasone (PVd) or daratumumab, pomalidomide, and dexamethasone (DPd). PVd or DPd were used as bridging therapy before starting CAR-T, and the dose of CAR-T used was 0.75 × 10^6^ cells/kg. The overall response was 84.6% in the cilta-cel group and 67.3% in the standard care group, with an odds ratio of 3.0 (95% CI: 1.8-5.0) (odds ratio >1 shows an advantage to cilta-cel). In 12 months' time, the progression-free survival in the cilta-cel group was 75.9%, while the standard care was 46.8%. Moreover, more patients had a complete response in the cilta-cel group compared to the standard care group, with percentages of 73.1% and 21.8%, respectively, and an odds ratio of 10.3 (95% CI: 6.5-16.4) [39].

Another phase 3 trial by Rodriguez-Otero et al. has also investigated the progression-free survival when using CAR-T (idecabtagene vicleucel, or ide-cel) compared to standard treatment. For the ide-cel group, the 12-month progression-free survival rate was 55%, and 30% for the standard treatment group. Furthermore, the ide-cel group had a higher percentage of patients with partial response or better than the standard treatment group: 71% (95% CI: 66-77) for the ide-cel group, and 42% (95% CI: 33-50) for the standard treatment group, with an odds ratio of 3.47 and a p-value of <0.001 [42].

A study by Alsdorf et al. compared the efficacy of cilta-cel to other treatment lines to treat relapsed refractory MM. Cilta-cel was used in the CARTITUDE 4 phase 3 randomized controlled trial (RCT), where cilta-cel was compared with PVd or DPd. The trial results were also compared to other trials that used standard treatment to treat relapsed/refractory MM, such as the CASTOR, CANDOR, and APOLLO RCTs. The comparison was made to assess the ORR and the progression-free survival. Regarding the cilta-cel group, the ORR was 89.7%, while in the other treatment groups, the ORR ranged from 42.4% to 79.6%. In addition, compared to all the treatment options, cilta-cel demonstrated a higher percentage of patients who achieved a complete response. In the cilta-cel group, the complete response was 78.1%, while in the other groups, the complete response ranged from 2.2% to 26.5%. All the aforementioned results are statistically significant, defined as a p-value of <0.01 [43].

Approved CAR-T Therapies

Two main therapies are approved by the FDA for the management of relapsed/refractory MM, which are cilta-cel and ide-cel [8,44]. However, there are other approved therapies included within the FDA criteria that treat other malignancies. In 2021, the FDA also approved two distinct CAR-T therapies to treat patients diagnosed with ALL, which include tisagenlecleucel (tisa-cel) and brexucabtagene autoleucel (brexu-cel). These two therapies usually treat refractory or relapsed cases of ALL in a specific age group of young adults, starting from the age of 18 to 25 years [45].

Refractory/relapsed cases of large B-cell lymphomas (LBCLs) were also studied to be treated with FDA-approved CAR-T therapies, such as tisa-cel, axicabtagene ciloleucel (axi-cel), and lisocabtagene maraleucel (liso-cel) [46,47]. A phase 2 study showed that 101 patients were treated with axi-cel; most of those patients had de novo diffuse large B-cell lymphoma (DLBCL), with the highest percentage of 76%, followed by transmuted follicular lymphoma (FL), and the lowest had primary mediastinal large B-cell lymphoma (PMBCL), which accounted for 16% and 8%, respectively. Axi-cel is only indicated to be used if two lines of systemic therapies have failed [46]. Despite that, 268 patients received liso-cel. It was used to treat patients with aggressive forms of the disease: 26% of them had ≥4 prior lines of systemic therapy, 34% had gone through auto-HSCT, 3% had allo-HSCT, 67% were refractory to chemotherapies, and finally, 44% never fulfilled a complete remission (CR), whereas 59% needed bridging therapy [47].

The phase 1b/2 CARTITUDE-1 trial was a 16-center study in the USA that included patients with diagnosed MM as per the IMWG criteria. The patients received several prior treatment lines, including PIs, IMiDs, and anti-CD38 antibodies. In the trial, they were given cilta-cel to assess safety and overall response as the primary endpoints, and progression-free survival and duration of response as the secondary endpoints. Initially, 113 patients were enrolled in the trial; however, 16/113 were not able to receive the cilta-cel infusion due to disease progression, death, or withdrawal from the study. The dose used was 0.75 × 10^6^ CAR-T cells per kg. The results were very promising: in 12 months, the ORR was 97% (95% CI: 91.2-99.4), and most patients responded to treatment within one month of infusion. Most patients (67%) showed a stringent complete response, and the 12-month progression-free survival was 77% (95% CI: 66.0-84.3). During the study, 14 deaths occurred, and they were mostly attributed to treatment effects such as cytokine release syndrome (CRS), sepsis, and infections. The results showed improvement after receiving cilta-cel by a median time of one month [40].

Ide-cel, also known as bb121, is also a BCMA-targeted therapy. In multiple studies, the ORR was high for all patients, and many of them had a stringent complete response [48]. The response rate was dose-dependent in several studies, meaning an increased dose of CAR-T leads to a better response. In a phase-1 trial, a dose of at least 150 × 10^6^ CAR-T cells showed a complete or partial response; the same was seen with the KarMMa trial, involving Japanese and non-Japanese cohorts, using a dose of 450 × 10^6^ CAR-T cells [41,48]. This was also supported by a retrospective analysis, which proved that the greater expansion of CAR-T cells is associated with a longer progression-free survival [49].

When comparing cilta-cel and ide-cel, although they have the same targets, there is a minor difference in structure between the two, which is in the binding domains. Cilta-cel has two binding domains for BCMA, while ide-cel has only one domain. This change in structure can significantly affect the efficacy. In a trial comparing ide-cel and cilta-cel involving patients with similar characteristics and similar prior treatment regimens, cilta-cel was found to be more effective. A greater number of patients achieved a complete response in the cilta-cel group as compared to ide-cel, in addition to a shorter duration to respond to therapy and reduced mortality rates. In terms of progression-free survival, the cilta-cel group had approximately 22.8 months, while the ide-cel group had only 9.8 months [41].

Ongoing Research on CAR-T Therapies

Currently, numerous phase 1/2a clinical trials are assessing the safety and efficacy of CAR-T therapies for MM by recruiting diverse patient populations, such as the CARTIFAN-1, anti-GPRC5D CAR-T cells therapy, and more [50,51]. This research aims to identify the responses of CAR-T in different populations, which can provide useful insights on the efficacy rates in different ethnic and cultural groups. In the CARTIFAN trial, testing cilta-cel on Chinese patients has shown that the efficacy is similar to previously conducted trials; however, there were increased rates of infections, hepatitis B virus (HBV) in particular. This was attributed to the high prevalence rates of HBV infection in China [50].

Apart from CAR-T cells treating MM, there are other benefits of using this line of therapy in managing other diseases, two of which are breast and ovarian cancers. A type of human epidermal growth factor receptor-2 (HER-2)-specific CAR-T cells was developed to target only HER-2+ cases of the disease. The research article showed that the innovative therapy killed the HER-2 cancerous cells ex vivo; however, it also activated the regression of the breast cancer cells in vivo [52].

A different type of HER-2-specific CAR-T cell was designed with an intracellular costimulatory domain of 4-1BB (HER2-BBζ), which showed promising results in treating breast cancer metastasis involving the brain. The mode of delivery was intraventricular, thus treating multifocal lesions [53]. 

Since CAR-T is an emerging therapy, the long-term side effects aren't known yet. A French registry known as DESCAR-T has been created to record the list of French patients who underwent CAR-T therapy; this will help understand the effects CAR-T has on the body, in addition to predicting the outcomes and risk factors associated with relapse [54].

Another ongoing clinical trial is CARTITUDE-6, which aims to compare the efficacy of using daratumumab and hyaluronidase, bortezomib, lenalidomide, and dexamethasone followed by cilta-cel (dose: 0.75 × 10^6^ CAR-T cells/kg) vs. followed by autologous stem cell transplant as a treatment for newly diagnosed MM. The results of the study have not been published yet [55].

Safety Profile of CAR-T

In most clinical trials and studies using CAR-T, CRS was the most frequently encountered adverse effect, followed by cytopenias and other hematological and immunological toxicities. CRS has multiple stages depending on its severity, initially starting with a fever for several days in most cases and then progressing to organ dysfunctions that can possibly lead to death [9,11,26]. In most patients, fortunately, CRS is not severe and is often graded 1 or 2, which can be successfully managed by tocilizumab. Sometimes, the addition of steroids and anakinra is advised. These treatment options are initiated after other causes of fever, such as sepsis and infusion reactions, are ruled out. Moreover, adding these medications along with CAR-T does not affect its efficacy [6,33,40]. Immune effector cell-associated neurotoxicity syndrome (ICANS) was also mentioned; it was most commonly seen following CRS. Its management is the same; furthermore, managing CRS with high doses of corticosteroids can prevent the occurrence of ICANS [31].

Respiratory tract infections, including pneumonias, were the most common types of infections reported in the CARTITUDE phase 1b/2 study and other similar setting studies. Most of these infections were graded as 3 or 4 [5,40].

In a study by Garfall et al., in which they tested the safety profile of CAR-T early in the progression of MM, they noticed that CRS was milder compared to patients with relapsed/refractory MM, which suggests that earlier initiation of CAR-T can reduce adverse effects and provide better outcomes [12]. Since CAR is a synthetic protein, it can be obtained from murine cells; however, this might result in adverse events such as rejection, since it is a foreign body and can reduce the number of CAR-T cells. Nevertheless, this can be resolved by using humanized CAR cells [6].

However, a study by Yamamoto et al. revealed that among the 33 consecutive patients, side effects such as cytopenias (neutropenia in 85% of patients, leukopenia in 58% of patients, anemia, and thrombocytopenia having equal percentages of 45%), specifically of grades 3 and 4 type, were also common. Patients receiving combination therapy were highly susceptible to side effects [56]. Those include early high-grade fevers and, rarely, sepsis, ICANS, and, lastly, death in one patient due to COVID-19. Lower doses during earlier progression of the disease also proved lesser toxicity levels [12].

Moreover, another study showed that decreasing the chemotherapy doses, such as melphalan, in patients who underwent autologous stem cell therapy (ASCT) from the standard 200 mg/m^2^ dose to 140 mg/m^2^ results in lower chances of experiencing renal insufficiency and cytotoxicity [37]. This raises concerns, as chemotherapy types and doses will also affect the patient's response. Table 5 demonstrates the different side effects encountered along with their frequencies [32].

**Table 5:** Commonly reported side effects of CAR-T according to the National Cancer Institute Table credit: [32] CAR-T, Chimeric antigen receptor T-cell

Side effect	Frequency
Cytokine release syndrome	95%
Infections	59%
Low blood platelet count	41%
Low neutrophil count	30%
Nerve problems	26%
Low antibody levels	12%

In a qualitative study based on semi-structured interviews that involved 45 patients who received ide-cel treatment for relapsed/refractory MM, a high percentage of patients were generally satisfied with the treatment. They reported few to no side effects following infusion (78%), and around half of the patients reported improved overall well-being after treatment (51%). In addition, patients were pleased that they did not need to follow a maintenance therapy, such as chemotherapy [57].

Cost

Till today, CAR-T is the most expensive therapy for MM; the cost ranges between $500,000 and $1,000,000. Its cost can be justified due to the complex production mechanism, the need for expensive manufacturing raw materials like viral vectors, and limited production agents [56,58]. Yamamoto et al. analyzed the cost-effectiveness of CAR-T and established that, although the starting cost of CAR-T is at the extreme end and is associated with several adverse events, it is more affordable in the long run and ensures a better quality of life for patients. Therefore, this emphasizes the need to compare long-term benefits versus initial costs [56]. 

Future recommendations

Some articles have listed possible correlations between radiation, infections, and other factors that could be causes of MM; however, none have been confirmed. Possible research ideas include conducting retrospective reviews, examining the presence of these factors, and investigating whether a certain risk factor is more pronounced in MM than the others.

In the future, further RCTs that compare the efficacy, survival rates, and safety profile of CAR-T therapy to the existing treatment lines are needed to prove its superiority. Although the price of CAR-T appears to be high initially, it may be cheaper in the long run; therefore, it is crucial to analyze its cost-effectiveness. This is a potential research topic to be discussed, as the literature regarding this matter is limited.

Due to the observed variance in treatment responses between patient groups with BCMA-expressing myeloma cells and those without, it is important to conduct more studies, including larger cohorts of patients, to reach a definite conclusion on whether BCMA has a role in treatment efficacy. This will help determine if the expression of BCMA impacts the effectiveness of CAR-T, creating more personalized treatment plans for patients with MM. 

Other than the generations of CAR-T, there are studies that illustrated the presence of fifth-generation CAR-T; however, limited research has been done on it, which necessitates further research. Since CAR-T is an emerging therapy, the long-term side effects are not known; therefore, retrospective studies might help investigate this matter initially, but the need for prospective studies persists to understand if there are any long-term adverse effects of CAR-T therapy that should be focused on. These studies are essential to guarantee patients' safety and to prepare for any undesired consequences.

So far, CAR-T is only utilized in patients who are heavily pretreated using various treatment methodologies. Although most of these studies proved significant improvements in these patients regardless of previous treatment lines, it is important to measure the efficacy of CAR-T alone. One study has confirmed that using CAR-T early on in the treatment led to successful outcomes and reduced adverse effects; therefore, more similar studies are required to further emphasize this point to improve patients' outcomes.

There are only a handful of studies that investigate the effectiveness of CAR-T over already existing therapies; therefore, RCTs can be done to investigate the effectiveness, compare the safety profile, survival rates, and more. Given that CAR-T is associated with several adverse events, future studies can focus on generating newer combination therapies in addition to CAR-T to reduce the occurrence of these adverse effects, leading to enhanced life expectancy and better overall well-being.

## Conclusions

CAR-T therapy is a rapidly emerging therapy with promising results, offering hope to patients who have limited therapeutic options. Despite its favorable outcomes, the cost, manufacturing techniques, and potential toxicity limit its use, which is an area of concern that should be addressed. 
